# Evolutionary Predictors of Post-COVID Syndrome

**DOI:** 10.3390/jcm15145550

**Published:** 2026-07-15

**Authors:** Maria Luisa Asensio Tomás, Philip Erick Wikman-Jorgensen, Jose Antonio Quesada-Rico, José Miguel Seguí-Ripoll, Vicente Gil-Guillén, Vicente Giner-Galvañ

**Affiliations:** 1 Internal Medicine Department, San Juan de Alicante University Clinical Hospital, 03550 San Juan de Alicante, Spain; 2 Fundación para el Fomento de la Investigación Sanitaria y Biomédica (FISABIO), 46020 Valencia, Spainginervicgal@gmail.com (V.G.-G.); 3 Infectious Diseases Unit, San Carlos Clinical University Hospital, 28040 Madrid, Spain; 4 Department of Clinical Medicine, Miguel Hernández University of Elche, 03202 Elche, Spain; vte.gil@gmail.com; 5 Internal Medicine Department, Virgen de los Lirios University General Hospital, 03804 Alcoy, Spain; 6 Medicine Area, Department of Community Nursing, Preventive Medicine and Public Health and History of Science Health, University of Alicante, 03690 San Vicente del Raspeig, Spain

**Keywords:** persistent COVID, post-COVID syndrome, PCFS, symptomatic association

## Abstract

**Objectives**: To analyze evolutionary predictors of post-COVID syndrome (PCS). **Methods**: This Cohort study was conducted from April 2021 to December 2023. PCS persistence was defined as a score of ≥2 points on the post-COVID-19 Functional Status (PCFS) scale (range 0–4, where higher scores indicate more limitations). Symptom clusters were identified using correspondence analysis. The association between PCS persistence and symptoms and symptom clusters were assessed using multivariable analysis, considering both the acute infection period and the baseline PCS visit variables. **Results**: The 112 included patients showed a mean of 6.1 symptoms (standard deviation [SD] 3.2), most commonly dyspnea (67.9%), asthenia (67.0%), fatigue (57.1%), and myalgia (49.1%). Regarding baseline functional status, 59.8% scored 2 points on the PCFS; 40.2%, 3 points; and 2.6%, 4 points. Moreover, 68.7% had depression and 83.0%, anxiety. Two symptom clusters emerged: anxiety + depression + severe PCFS, and palpitations + hyporexia + dyspnea. At a mean of 12.1 months (SD 9.2), the persistence rate was 33.9% (cumulative persistence time 20 months [SD 11.9]). Persistence was significantly associated with the absence of rhinitis (odds ratio [OR] 3.67, 95% confidence interval [CI] 1.28–10.52) during acute infection, and the presence of fatigue (OR 4.88, 95% CI 1.77–13.44) and depression (moderate: OR 4.28, 95% CI 1.05–17.44; severe: OR 10.31, 95% CI 2.60–40.90) at the baseline visit. The model multivariable demonstrated a good fit (likelihood ratio test 30.1, *p* < 0.001) and predictive capacity (area under the receiver operating curve 0.804). The anxiety + depression + severe PCFS cluster was significantly associated with persistence (OR 3.26, 95% CI 1.21–8.76; *p* = 0.012). **Conclusions**: A third of patients with PCS still experience significant functional impairment 20 months after onset. Persistence is associated with the absence of rhinitis during acute infection and with severe anxiety, depression, and functional impairment measured by the PCFS on detection of PCS.

## 1. Introduction

Post-COVID syndrome (PCS) is characterized by significant phenotypic heterogeneity and lacks a universally accepted clinical definition. This condition entails substantial functional impairment and poses a major public health challenge, with an estimated 6–26% of patients infected with SARS-CoV-2 developing symptoms that persist beyond the acute phase [1,2]. As of March 2026, the World Health Organization (WHO) has reported 281,757,395 cumulative infections within the European Region; based on current prevalence estimates, this translates to at least 17 million individuals suffering from PCS across the continent [3].

The WHO defines PCS as a constellation of signs and symptoms, which cannot be explained by an alternative diagnosis, persists beyond 12 weeks after infection with SARS-CoV-2, and last at least two months. Other organizations, such as the US Centers for Disease Control (CDC) or National Institute for Health and Care Excellence (NICE), differ in their definitions, establishing a symptom-free period of four weeks from the acute infection [4,5,6].

The challenge of reaching a standardized definition responds to the marked phenotypic heterogeneity of the syndrome, with up to 200 symptoms of varying intensity recorded across diverse series, along with a very high fluctuation over time and disparate outcomes [7,8,9]. However, shared clarity has emerged around the negative impact PCS has on daily function [10,11,12]. A controlled study with a two-year follow-up estimated that among outpatients, PCS contributed to the loss of 80.4 disability-adjusted life years (DALYs) per 1000 people [13]. In this regard, and in contrast to most symptom-based approaches, the National Academies of Sciences, Engineering, and Medicine consider the impact of symptom accumulation on functionality and quality of life in their definition, an approach which has also been adopted by our group [14,15].

The natural history and persistence of the process remain unknown. Female sex, age over 40 years, obesity, COVID-19 severity, smoking, pre-existing comorbidities, and low socioeconomic status have been proposed as risk factors, while vaccination appears to protect against PCS [16,17,18,19,20]. Information regarding its clinical course is scarcer. A few studies with a maximum follow-up of two years indicate that 20% to 30% of patients with mild COVID-19 experience persistent PCS [21,22,23]. Recovery time is still uncertain, and knowledge of predictive factors for clinical evolution is minimal [24]. Two recent studies identified low education level, unemployment, presence of symptoms similar to chronic fatigue syndrome, and upper respiratory and gastrointestinal symptoms as risk factors for persistence. However, these were cross-sectional, self-report studies [16,25].

In light of the gap in evidence and the important public health impact of PCS, this study aims to identify factors associated with PCS persistence in patients with significant secondary functional impairment. A secondary aim is to refine the definition of PCS through the search for phenotypic associations and their potential as evolutionary predictors.

## 2. Materials and Methods

### 2.1. Study Design

**Figure 1:**
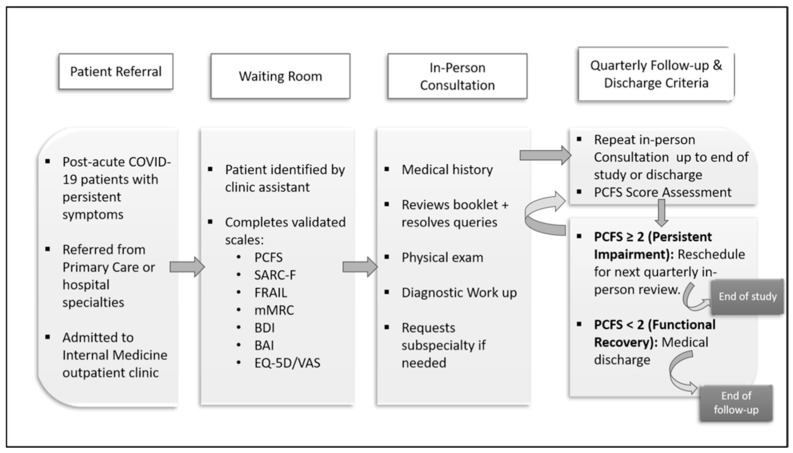
Flow diagram of the study procedures and participant follow-up.

This ambispective cohort study recruited patients referred to the PCS clinic of the Internal Medicine Department at the San Juan University Hospital (Alicante, Spain) from 1 April 2021 to 30 December 2022, following a specific departmental care protocol (A summary of the study protocol is available in Supplementary Material S1) [15]. Data from April 2021 to January 2022 were analyzed retrospectively, whereas data collection from 26 January 2022, onwards was conducted prospectively following formal approval by the Ethics Committee of San Juan Hospital on 26 January 2022. Patients were followed quarterly in person by the attending consultant until recovery or the end of the observation period on 30 December 2023. Assessments combined patient-completed validated scales PCFS, Strength, Assistance in walking, Rise from a chair, Climb stairs, and Falls scale (SARC-F), Fatigue, Resistance, Ambulation, Illnesses, and Loss of weight escale (FRAIL), modified Medical Research Council dyspnea scale (mMRC), Beck Depression Inventory (BDI), Beck Anxiety Inventory (BAI), and the EuroQol 5-dimension scale/Visual Analogue Scale (EQ-5D/VAS), which included brief written instructions at the beginning of each questionnaire with physician-verified clinical evaluations [26,27,28,29]. Patients with functional recovery (PCFS < 2) were discharged to primary care, whereas those with persistent impairment (PCFS ≥ 2) continued quarterly reviews. The detailed workflow and a summary of the study protocol are available in Figure 1 and Supplementary Material S1, respectively. The study was approved by the Ethics and Research Committee of the San Juan de Alicante Hospital on 26 January 2022 (Code: 22/006).

### 2.2. Participants

We consecutively included all patients over 18 years of age with persistent symptoms lasting more than four weeks after microbiologically confirmed SARS-CoV-2 infection (antigen test and/or polymerase chain reaction), regardless of hospital admission status, following signed informed consent. Participants were required to have significant secondary functional impairment, defined as a baseline post-COVID-19 Functional Status (PCFS) scale score of 2–4 points (range 0–4, with higher scores indicating more functional limitations). Patients were excluded if symptoms were attributable to an alternative diagnosis.

### 2.3. Variables

The primary outcome variable was symptom persistence, defined as a PCFS score of 2 or more, which was assessed quarterly at each follow-up visit. Explanatory variables collected from electronic health records included demographic data, medical history, comorbidities (Charlson Comorbidity Index) at the time of COVID-19 diagnosis, acute episode symptoms and severity (requirement for oxygen therapy or intensive care unit admission), and vaccination status (full vaccination: >21 days post-second dose versus unvaccinated/incomplete vaccination). Other explanatory variables were collected at the baseline PCS visit, including persistent symptoms based on a list of the 17 most frequent symptoms reported in the PCS literature (Supplementary Material S2). Patients were provided with a self-administered booklet with different scales: modified Medical Research Council (mMRC) scale to quantify dyspnea (0–4 points); Beck Depression Inventory (BDI) to assess depression and its severity (absent, mild, moderate, or severe); Beck Anxiety Inventory (BAI) to assess anxiety and its severity (Minimal, mild, moderate, or severe); SARC-F scale to detect sarcopenia (>4 points); FRAIL scale for frailty assessment; EuroQol-5D (EQ-5D) to measure health-related quality of life (0–100); and the Post-COVID-19 Functional Status (PCFS) scale to assess the global functional daily impact (0: no functional limitations; 1: negligible limitations; 2: slight limitations; 3: moderate limitations; 4: severe limitations/nursing care dependency). All data were managed using REDCap electronic data capture tools (Vanderbilt University, Nashville, TN, USA) [30].

### 2.4. Statistical Analysis

An initial descriptive analysis was performed, presenting categorical variables as frequencies and quantitative variables as mean, median, and standard deviation (SD). Persistence at the end of follow-up was compared according to explanatory variables and expressed as odds ratios (OR) and 95% confidence intervals (CI). Due to the large number of variables in the study a preselection of predictors was performed based on their significant association (*p* < 0.10) in the crude fit. Using this preselection, a stepwise variable selection was performed based on the Akaike Information Criterion (AIC), and a final set of variables was included in a multivariable logistic regression model. Goodness-of-fit was assessed using the likelihood ratio test (LRT), and predictive capacity using the area under the receiver operating characteristics curve (AUC). Multiple correspondence analysis was conducted to analyze similarity patterns among individuals according to baseline variables [31]. After determining the optimal number of dimensions using the Biplot method, graphical representations of the variable categories were constructed within the selected dimensions. In these plots, variable categories with similar profiles tend to cluster together, while negatively correlated categories are positioned in opposing quadrants. The distance from each category’s position to the origin (0,0) measures the quality of that category’s representation within the dimension, such that categories further from the origin are better represented. The quality of representation for these categories across dimensions was based on the squared cosine (Cos2), where 1 represents the highest quality and 0 the lowest.

All statistical analyses were performed using IBM SPSS^©^ Statistics software, version 28.0 (IBM Corp., Armonk, NY, USA) [32].

## 3. Results

### 3.1. Sample Characteristics

Of the 183 patients referred to the PCS outpatient clinic, 68 (37.2%) were excluded prior to enrollment: 46 (25.1%) had a PCFS score of 0–1, 21 (11.5%) showed symptoms attributable to alternative diagnoses, and 1 (0.5%) had symptoms secondary to vaccination. Consequently, a total of 115 patients were formally recruited for the study. Of these, 3 (1.6%) were subsequently lost to follow-up, resulting in a final sample of 112 patients. The baseline clinical characteristics of the sample are summarized in Table 1. Mean age was 51.2 (SD 13.2) years. Most participants were women (78.6%) and aged 40–64 years (74.1%). Regarding baseline comorbidities, 13.4% were smokers, and 14.3% had at least one underlying chronic disease, most commonly dyslipidemia (18.8%), hypertension (17.9%), anxiety, and depression (13.4% each). The burden of comorbidities was quite low, with a mean Charlson Comorbidity Index of 0.2 (SD 0.6); just two participants (1.8%) showed high comorbidity. A third of the participants had completed the full SARS-CoV-2 vaccination schedule. A post hoc chi-square analysis evaluating vaccination status across age groups revealed that older individuals (age ≥ 65 years) had a significantly higher rate of complete primary vaccination coverage compared to younger cohorts (approximately 75% vs. 30%, respectively; *p* = 0.012).

### 3.2. Acute Infection

During participants’ acute COVID-19 infection, the primary symptom was cough (71.4%), followed by fever (64.3%), dyspnea (63.4%), asthenia (62.5%), myalgia (60.7%), and headache (56.3%) (Table 2). The mean number of acute symptoms per patient was 5.7 (SD 3). Hospitalization was required for 18.8% of patients, with 18.8% and 3.0% of the total cohort requiring supplemental oxygen therapy or ICU admission, respectively.

### 3.3. Post-COVID Syndrome Evolution

At the baseline visit, the most frequent PCS-related symptoms were dyspnea (67.9%), asthenia (67.0%), fatigue (57.1%), and myalgia (49.1%), with a mean of 6.1 symptoms per patient (SD 3.2) (Table 3). Regarding baseline conditions most participants exhibited slight (59.8%) or moderate (40.2%) functional impairment as measured by the PCFS, with only three cases (2.6%) exhibiting severe impairment. The mean EuroQol-5D health-related quality of life visual analog scale score (EQ-VAS) was 52.1 out of 100 (SD 16.8). The prevalence of sarcopenia (SARC-F) was 43.8%, and 15.1% met the criteria for frailty according to the FRAIL scale. According to the mMRC scale, 7.1% of the participants presented grade 0 dyspnea, 32.1% grade 1, 20.5% grade 2, and 40.2% grades 3–4. Finally, depression was present in 68.7% of the participants (mild 10.7%, moderate 28.6%, severe 29.5%), and anxiety was detected in 83.0% (mild 19.6%, moderate 20.5%, severe 42.9%).

**Figure 2:**
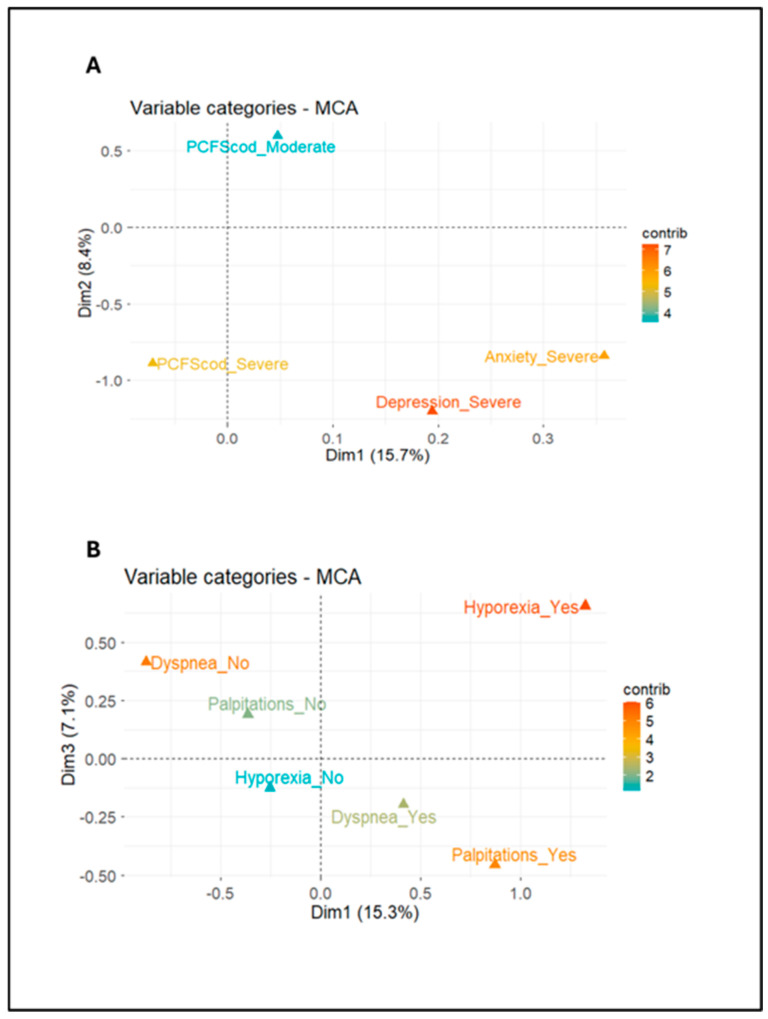
Analysis of phenotypic categories using multiple correspondence analysis. (**A**) Graph of the categories of the variables that contribute most to the construction of dimensions 1 and 2 (Cos2 > 0.4). (**B**) Graph of the categories of the variables that contribute most to the construction of dimensions 1 and 3 (Cos2 > 0.4). The dashed lines represent the coordinate axes intersecting at the origin (0,0).

On multiple correspondence analysis, three dimensions explained 30.1% of the variability, and two symptom clusters were identified (Figure 2). Dimensions 1 and 2 (Figure 2A) showed a similar profile among patients with anxiety, depression, and severe functional impairment (PCFS). Dimensions 1 and 3 (Figure 2B) showed that patients with hyporexia, palpitations, and dyspnea shared a similar profile compared to those without these symptoms.

Mean follow-up was 12.1 months (SD 9.2). At the end of the follow-up period, 38 patients (33.9%) showed symptom persistence (PCFS ≥ 2), with a mean cumulative symptom duration of 20.7 months (SD 11.9).

### 3.4. Risk Factors Associated with Persistence

When evaluating individual symptoms in relation to both the acute infection period and the baseline PCS visit, the crude analysis (Supplementary Material S3) showed that factors significantly associated with a higher probability of persistence were mainly evident at the baseline PCS time point. These factors were: severe anxiety (OR 21.3, 95% CI 2.63–170.3), severe depression (OR 8.23, 95% CI 2.37–28.6), moderate anxiety (OR 7.87, 95% CI 0.87–71.13), moderate depression (OR 5.30, 95% CI 1.51–18.65), fatigue (OR 3.59, 95% CI 1.50–8.62), moderate/severe PCFS (OR 3.05, 95% CI 1.36–6.86), and asthenia (OR 3.02, 95% CI 1.18–7.75). Finally low EQ-VAS scores (OR 0.97, 95% CI 0.95–0.99), indicating a 3% increase in persistence risk for every point lost on the self-rated health scale. Notably, neither hospitalization, acute corticosteroid use, nor the time from acute infection to the baseline visit significantly influenced the final outcome, yielding odds ratios of 1.25 (95% CI 0.47–3.34; *p* = 0.66), 1.41 (95% CI: 0.56–3.52; *p* = 0.47), and 1.04 (95% CI 0.98–1.09; *p* = 0.21), respectively. Similarly, no significant differences were observed regarding sex (OR 1.03, 95% CI 0.40–2.69; *p* = 0.95) or age across any of the analyzed brackets (*p* > 0.05).

Table 4 presents the multivariable logistic regression model. Six predictors were preselected in the crude fit (8 parameters), and four significant variables were included in the final model (8 parameters). Factors independently associated with symptom persistence at the end of the follow-up were: absence of rhinitis during acute infection (OR 3.67, 95% CI 1.28–10.52) and fatigue (OR 4.88, 95% CI 1.77–13.44), moderate depression (OR 4.28, 95% CI 1.05–17.44), or severe depression (OR 10.32, 95% CI 2.60–40.90) at baseline. The model demonstrated a good fit (LRT 30.1; *p* < 0.001) and strong predictive capacity (AUC 0.804). The AUC estimated by internal validation with 1000 bootstrapping simulations is 0.745 with 95% CI (0.658–0.838).

Table 5 summarizes the multivariable analysis of the identified symptom clusters. Unlike the cluster comprised by hyporexia, palpitations, and dyspnea, which was not associated with persistence, the cluster of depression, anxiety, and severe PCFS (grade 4) was significantly associated with persistence at the end of study (OR 3.26, 95% CI 1.20–8.76; *p* = 0.019).

## 4. Discussion

At one-year follow-up, over a third of patients with PCS exhibited persistent and significant functional impairment. Taken individually, associated risk factors were the absence of rhinitis during acute SARS-CoV-2 infection, along with fatigue and moderate or severe depression at the baseline PCS assessment. Consistently, the symptom cluster comprised by anxiety, depression, and severe PCFS was associated with persistence, particularly when these symptoms were severe.

The demographic characteristics and clinical presentation of PCS in the present series are consistent with existing literature, showing a marked predominance of middle-age female patients (78.6%), which likely reflects that female sex is a major risk factor for PCS. However, regarding the long-term evolution of the syndrome, while recent literature suggests that women experience a higher symptom burden and a lower rate of recovery at one year compared to men, our findings revealed no statistically significant sex-related differences in symptom persistence after 12 months of follow-up [33]. This finding should be interpreted cautiously, as the marked imbalance between women and men in our series limits statistical power to detect sex-related prognostic effects.

The most frequent symptoms were dyspnea (67.9%), asthenia (67.0%), fatigue (57.1%), and myalgia (49.1%), alongside a high prevalence of anxiety (83%), depression (68.7%), and impaired quality of life. This case series aligns with previous reports, despite variations in prevalence across different cohorts. For example, in a recent study of 334 patients, Kosowan et al. found that the most common symptoms were fatigue (79%), brain fog (76%), dyspnea (65.3%), and headache (64.1%) [34]. Conversely, a meta-analysis of 32 cohort studies including 10,950 patients, reported weakness (41%), malaise (33%), fatigue (31%), brain fog (26%), dyspnea (25%), and reduced quality of life (37%) as the most prevalent manifestations [35].

The literature is generally consistent on the symptoms of PCS, but their prevalence varies due to heterogeneous study designs, populations, measurement tools, and the definition of the syndrome itself [4]. PCS is characterized by a high symptoms burden—in this study, a mean of six per patient—and the subjective nature of most of them. This aligns with the high rates of anxiety and depression detected in our study, whose relevance stems from their demonstrated association with persistence. In this regard, the significance of the phenotypic cluster “anxiety + depression + severe PCFS” as a risk factor for persistence underscores the potentially central role of psychological manifestations in PCS and its persistence.

The predictive model found in the present cohort has direct clinical applicability for post-COVID outpatient clinics. Main predictors like fatigue, depression, and PCFS score are easily assessed simply applying low-cost, validated scales, enabling rapid risk-stratification and early multidisciplinary support (especially for the high-risk anxiety–depression–impairment cluster). While the model demonstrates robust performance (AUROC 0.804), external validation in larger cohorts remains essential before widespread clinical implementation to ensure it effectively optimizes healthcare resources.

After over a year of follow-up, approximately one-third (33.9%) of the patients exhibited PCS persistence, defined as a score of two or more on the PCFS scale. A slightly higher persistence rate (43%) at two years’ follow-up was observed in a previous study using a similar, functional impact criterion, specifically the inability to perform routine work tasks [12]. In contrast, other studies report persistence rates as high as 92.4% for a similar follow-up period [36]. The reason for these discrepancies resides, once again, in the heterogeneous definitions of “persistence.” Our study shows a persistence rate similar to that reported by Frallonardo et al., likely because both applied functional criteria, albeit different ones, whereas Mateu et al. defined persistence as the continuation of any PCS-related symptoms. Had the latter definition been applied in our study, the final prevalence would undoubtedly be higher. Our patients experienced a mean duration of persistence of 20.7 (SD 11.9) months, representing a significant individual and social disease burden in terms of functional loss. The chronic and persistent nature of PCS, beyond one year in most cases, is supported by a recent review showing that 71% of people reporting long COVID had persistent symptoms for at least one year, 51% for at least two years, and 31% for at least three years [37]. It has also to be considered that while clinical symptoms may naturally fluctuate over time, the time elapsed from acute infection to baseline assessment in the present study probably did not significantly influence outcomes (*p* = 0.21), as potential observation biases were reduced via systematic quarterly reviews.

When analyzing individual variables associated with PCS persistence, previous studies have established links with various factors related to acute infection, such as disease severity [37]. In contrast, our analysis supports an association only for the variable “absence of rhinitis.” Most predictive factors were instead related to manifestations at the baseline PCS assessment, specifically the presence of fatigue and moderate or severe depression. Consistent with these finding, the “anxiety + depression + PCFS” phenotypic cluster was significantly associated with persistence, especially when each individual variable showed severe grades. This stands in contrast to the other phenotypic cluster identified in the correspondence analysis, “palpitations + hyporexia + dyspnea”, which showed no such association.

Frailty and functional vulnerability may also represent relevant prognostic domains in both COVID-19 and post-COVID-19 syndrome. Our group has previously demonstrated a high prevalence of malnutrition (49.5%), sarcopenia (32.7%), and frailt assessed via the FRAIL scale (28.7%) among hospitalized patients with acute COVID-19. Furthermore, these clinical domains exhibited a strong correlation with in-hospital prognosis [38]. In the present cohort, frailty was also systematically assessed using the FRAIL scale, with 15.1% of participants meeting frailty criteria at the baseline visit. Although frailty was not identified as an independent predictor in our multivariate model, this finding should be interpreted cautiously given the relatively young age, low comorbidity burden, and limited number of frail participants in the sample. Recent evidence supports the prognostic relevance of frailty in COVID-19, although its relevance may differ according to the pandemic waves and individual vaccination status. Siniscalchi et al. showed that the Clinical Frailty Scale stratified hospital mortality risk among vaccinated patients during the fourth wave, whereas it was less informative in unvaccinated patients from the first wave, suggesting that frailty may become particularly useful when acute disease severity and pandemic-related factors are less dominant determinants of the outcome [39]. Together with our finding that PCFS-defined functional impairment and the anxiety + depression + severe PCFS cluster were associated with persistence, these data support the need to incorporate frailty and functional vulnerability assessments into longitudinal PCS studies. Future studies with larger and older cohorts should clarify whether frailty acts as an independent predictor of PCS persistence, a mediator of poor recovery, or a marker of reduced physiological reserve.

Interestingly, the oldest age group (≥65 years) did not exhibit significantly higher odds for post-COVID persistence. This is likely explained by immunization patterns within our sample, as older participants had significantly higher rates of complete vaccination coverage than younger individuals. Given that early campaigns prioritized older populations in our country, this high coverage may have exerted a protective effect, minimizing the probability of persistence in this cohort.

The significance of not presenting rhinitis during acute infection has been previously reported in the literature [36]. This finding may reflect a diminished innate antiviral immune response in the nasal mucosa, the primary barrier against SARS-CoV-2 infection, potentially leading to impaired viral clearance [40]. Notably, persistent viral reservoirs are among the hypothesized pathogenic mechanisms underlying PCS. A key finding of our study is that mental health variables, such as anxiety and depression, whether analyzed independently or as part of the described phenotypic clusters, are the primary determinants of persistence.

Regarding depression, current evidence suggests that a history of depressive disorders is a risk factor for developing PCS; however, it had not been previously identified as a predictor of long-term persistence [19]. While this association might suggest that such psychological symptoms are reactive to the functional limitations imposed by PCS, a direct consequence of the viral infection and its known neurotropism cannot be ruled out. In support of this, studies using FDG-PET in PCS patients have demonstrated serotonin and dopamine depletion in the central nervous system, as well as frontal brain hypometabolism [41]. While establishing a direct causal link between SARS-CoV-2 neurotropism and these symptoms is limited by the lack of a non-infected control cohort, our data strongly point toward a de novo phenomenon. Specifically, the fact that the vast majority of patients presenting with depression (80.5%) and anxiety (86.0%) at the first visit had no prior history of this diseases strongly supports a genuine de novo manifestation, suggesting a significant post-COVID psychological burden.

A primary limitation of this study is its sample size, which is smaller than that of similar prospective cohorts and may have limited the statistical power to detect certain associations. Specifically, due to the high number of predictors relative to the number of events, the multivariate model may suffer from overfitting; thus, the magnitudes of these associations should be interpreted with caution. This is reflected in the slight optimism of the estimates, with an observed AUC of 0.804 dropping to an honest validation AUC of 0.745. Consequently, potential confounders inherent to the acute phase were excluded from the final multivariable models due to a lack of statistical significance in univariate analyses or insufficient subgroup sizes. These variables included acute corticosteroid use, the time from acute infection to baseline assessment, and acute-phase severity such as hospitalization. Furthermore, the very small subsets of patients who received antivirals or required ICU care limit our ability to definitively evaluate the impact of critical illness. Future studies with larger sample sizes are therefore required to confirm these findings.

Nevertheless, some uncollected variables may have influenced the outcomes. For instance, the different SARS-CoV-2 variants along the inclusion period (wild-type, Alpha, Delta, and Omicron) could have acted as confounders, as Omicron has been associated with milder PCS phenotypes [42,43]. Regarding vaccination status, our protocol did not record the administration of booster doses or the exact time elapsed since the last vaccine dose, which may introduce confounding due to waning humoral immunity. Interestingly, recent systematic evidence suggests that booster doses might not drastically alter long-term outcomes, finding no statistically significant protective effect against post-acute COVID-19 syndrome (RR: 0.66; 95% CI: 0.41–1.09) [43]. In light of these findings, while the lack of this data represents a limitation, it is highly possible that its inclusion would not have changed our main conclusions. Furthermore, socioeconomic status, educational levels, and pharmacological treatments administered during follow-up were not recorded, thereby limiting our ability to evaluate their potential confounding influence on long-term recovery trajectories.

Conversely, the prospective design and the systematic collection of variables using validated scales ensure the robustness of our findings, as evidenced by the overall consistency of the results. Furthermore, the use of easily applicable clinical scales allows our model to be utilized for monitoring post-COVID-19 syndrome (PCS) evolution in daily clinical practice, representing another key strength of the study.

## 5. Conclusions

The high prevalence of patients with elevated scores on the post-COVID-19 Functional Status (PCFS) scale confirms the substantial functional impact of PCS. Our findings suggest progression to chronicity in a significant percentage of patients, with primary predictors appearing to be independent of the acute infection phase. In contrast to previous studies, these predictors are predominantly psychological in nature; however, it remains unclear whether these factors are reactive to profound functional limitations or a direct consequence of the initial CNS viral insult.

In the context of existing literature, this study underscores the relevance of psychological factors as the main result. This evidence suggests that active screening of anxiety and depression as well as functional status through the systematic use of validated scales could facilitate pharmacological and non-pharmacological interventions, potentially improving the clinical course of this syndrome, currently without an effective cure.

The marked heterogeneity of symptoms associated with PCS and the absence of universal diagnostic and monitoring criteria highlight the urgent need for standardized approaches. Without it, further research remains constrained. The inherently subjective nature of most clinical manifestations of PCS signals the potential need to adjust research strategies. To this end, this study applied a novel framework for characterizing the syndrome: rather than evaluating individual symptoms, we assessed the overall functional impact as a unifying metric. Additionally, the identification of significant symptom clusters may help refine the clinical definition by excluding manifestations that are likely not core components of the syndrome.

## Figures and Tables

**Table 1:** Participant characteristics (N = 112). SD: standard deviation.

Variable		Frequency
Age in years	Mean (SD)	51.2 (13.2)
	<40 years, n (%)	17 (15.2)
	40–64 years, n (%)	83 (74.1)
	≥65 years, n (%)	12 (10.7)
Women, n (%)		88 (78.6)
Comorbidities	Dyslipidemia, n (%)	21 (18.8)
	Hypertension, n (%)	20 (17.9)
	Smoking, n (%)	15 (13.4)
	Anxiety, n (%)	15 (13.4)
	Depression, n (%)	15 (13.4)
	Diabetes mellitus, n (%)	9 (8.0)
	≥1 chronic medical condition, n (%)	16 (14.3)
	Charlson comorbidity index, mean (SD)	0.2 (0.6)
SARS-CoV-2 vaccination	Complete, n (%)	38 (33.9)
	Incomplete or unvaccinated, n (%)	74 (66.1)

**Table 2:** Symptoms and treatment during the acute phase of COVID-19 (N = 112). ICU: intensive care unit.

Symptoms	n (%)
Cough	80 (71.4)
Fever	72 (64.3)
Dyspnea	71 (63.4)
Asthenia	70 (62.5)
Myalgia	68 (60.7)
Headache	63 (56.3)
Anosmia/ageusia	47 (42.0)
Hyporexia	38 (33.9)
Odynophagia	34 (30.4)
Rhinitis	34 (30.4)
Expectoration	26 (23.2)
Diarrhea	20 (17.9)
Chest pain	15 (13.4)
Arthralgia	12 (10.7)
Treatment	
Corticosteroids	25 (22.3)
Hospital admission	21 (18.8)
Oxygen therapy	21 (18.8)
Admission to intensive care unit	4 (3.6)

**Table 3:** Clinical characteristics PCS at baseline visit and on follow-up. PCFS: Functional status, SARC-F: Strength, Assistance with walking, Rise from a chair, Climb stairs, and Falls. FRAIL scale: Fatigue, Resistance, Ambulation, Illnesses, Loss of Weight. mMRC: Modified Medical Research Council dyspnea scale. EuroQol-5D (EQ-5D) VAS: Visual analogue scale of the Euro Quality of Life questionnaire (health-related quality of life).

Symptoms Present at Baseline Visit, n (%)		Frequency
Dyspnea		76 (67.9)
Asthenia		75 (67.0)
Fatigue		64 (57.1)
Myalgia		55 (49.1)
Headache		52 (46.4)
Brain fog		52 (46.4)
Insomnia		47 (42.0)
Arthralgias		41 (36.6)
Cough		35 (31.3)
Palpitations		33 (29.5)
Anosmia/ageusia		29 (25.9)
Chest pain		26 (23.2)
Hyporexia		18 (16.1)
Odynophagia		13 (11.6)
Diarrhea		10 (8.9)
Baseline conditions according to validated assessment scales		
Functional status (PCFS), n (%)	Grade 2: slight functional limitations	67 (59.8)
	Grade 3: moderate functional limitations	42 (40.2)
	Grade 4: severe functional limitations	3 (2.6)
Quality of life (EQ-5D-5L visual analog scale), mean (SD)		52.1 (16.8)
Sarcopenia (SARC-F), n (%)		49 (43.8)
Frailty (FRAIL), n (%)		17 (15.1)
Dyspnea (mMRC), n (%)	Grade 0	8 (7.1)
	Grade 1	36 (32.1)
	Grade 2	23 (20.5)
	Grade 3–4	45 (40.2)
Depression (Beck Depression Inventory), n (%)	None	35 (31.3)
	Mild	12 (10.7)
	Moderate	32 (28.6)
	Severe	33 (29.5)
Anxiety (Beck Anxiety Inventory), n (%)	Absent	19 (17.0)
	Mild	22 (19.6)
	Moderate	23 (20.5)
	Severe	48 (42.9)
Follow-up		
Months of follow-up, mean (SD)		12.1 (9.2)
Months since onset of PCS symptoms, mean (SD)		8.5 (7)
Persistence (PCFS grade ≥ 2), n (%)		38 (33.9%)
Months of symptom persistence, mean (SD)		20.7 (11.9)

**Table 4:** Multivariable logistic model for persistence of post-COVID symptoms (n = 112) OR: odds ratio. CI: confidence interval. Ref: reference category.

Variable	OR	95% CI	*p*
Age in years			
<40	1.49	(0.20–11.22)	0.70
40–54	1.10	(0.20–6.07)	0.91
55–64	1.15	(0.18–7.31)	0.88
≥65	Ref.		
No rhinitis during acute infection	3.67	(1.28–10.52)	0.015
Fatigue at baseline visit	4.88	(1.77–13.44)	0.002
Depression at baseline visit			
Absent	1		
Mild	3.22	(0.53–19.47)	0.20
Moderate	4.28	(1.05–17.44)	0.041
Severe	10.32	(2.60–40.90)	<0.001

**Table 5:** Age-adjusted multivariable logistic model for post-COVID symptom persistence (N = 112) for the symptom cluster “depression, anxiety and severe PCFS”. OR: odds ratio. CI: confidence interval. Ref: reference category.

Variable	OR	95% CI	*p*
Age in years			
<40	1.20	0.21–6.56	0.83
40–54	1.51	0.35–6.35	0.58
55–64	1.33	0.27–6.48	0.73
≥65	Ref.		
Cluster: anxiety + depression + severe PCFS	3.26	(1.20–8.76)	0.019

## Data Availability

The original contributions presented in this study are included in the article; further inquiries regarding the raw datasets can be directed to the corresponding author.

## Supplementary Materials

The following supporting information can be downloaded at https://www.mdpi.com/article/10.3390/jcm15145550/s1, S1. Clinical care protocol for patients with SPC at San Juan Hospital in Alicante. S2. List of most frequent symptoms. S3. Simple logistic regression model for persistence of post-COVID syndrome (N = 112).

## Abbreviations

The following abbreviations are used in this manuscript:

PCS	Post-COVID syndrome
SARS-CoV-2	type 2 coronavirus causing severe acute respiratory syndrome
WHO	World Health Organization
CDC	Centers for Disease Control
PCFS	post-COVID-19 Functional Status
DALYs	disability-adjusted life years
COVID 19	Disease caused by SARS-CoV-2
DLP	Dislipemia
m MRC	Modified Medical Research Council Scale
HBP	High blood pressure
IAB	Beck Anxiety Inventory
TBI	Beck Depression Inventory
DM	Diabetes Mellitus
EQ-5D	EuroQol-5D Health-Related Quality of Life Scale
