# Supplementary material for: Evolutionary Predictors of Post-COVID Syndrome
Source: J Clin Med. 2026 Jul 15;15(14):5550. doi: 10.3390/jcm15145550 (PMC13412679; doi:10.3390/jcm15145550)
Supplement: Supplementary file 1 [file jcm-15-05550-s001.zip › jcm-4403834-supplementary.pdf]

SUPPLEMENTARY MATERIAL

S1. *Summary Protocol for patients with SPC at San Juan Hospital in Alicante: Follow-Up Procedures*

1. Patient Referral

- ***Target Population:*** *Patients presenting with persistent clinical symptoms consistent with Post-COVID Syndrome following acute COVID-19 infection.*
- ***Referral Sources:*** *Primary Care Centers (Centros de Salud) and other hospital specialties.*
- ***Setting:*** *Outpatient Consultation Clinic, Department of Internal Medicine.*

2. Assessment Workflow

- ***Step 1: Arrival & Screening:*** *Scheduled patients are identified in the waiting room by the clinic assistant.*
- ***Step 2: Self-Administered Booklet:*** *The assistant provides patients with a standardized assessment booklet. This booklet includes an introductory guide with clear instructions.*
- ***Step 3: In-Person Consultation:*** *During the face-to-face visit, the* **attending consultant** *performs the following actions:*
  - *Takes a comprehensive medical history and reviews the booklet answers together with the patient, resolving any queries.*
  - *Conducts a physical examination, including oxygen saturation.*
  - *Performs 1-minute sit-to-stand test (1-min STST).*
  - *Reviews or requests follow-up laboratory blood tests or Chest X rays.*
  - *Refers the patient to other medical specialties if deemed necessary (e.g., Physical Medicine and Rehabilitation, Otorhinolaryngology, Psychiatry, etc.)*
- ***Step 4: Data Recording:*** *Verified outcomes are immediately recorded by the consultant into the patient's electronic clinical history.*

3. Summary of Included Assessment Scales

- ***Functional Status:*** *Post-COVID-19 Functional Status (PCFS) scale.*
- ***Sarcopenia & Frailty:*** *SARC-F scale and FRAIL scale.*
- ***Respiratory Symptoms:*** *Modified Medical Research Council (mMRC) dyspnea scale.*
- ***Psychological Evaluation:*** *Beck Depression Inventory (BDI) and Beck Anxiety Inventory (BAI).*
- ***Quality of Life:*** *EuroQol-5D (EQ-5D) and the Visual Analog Scale (VAS, 0–100).*

4. Follow-up Intervals and Discharge Criteria

- ***Frequency:*** *Assessments are repeated in person every 3 months following the exact workflow described above.*
- ***Discharge:***
  - ***PCFS score < 2:*** *The patient is informed of clinical improvement, discharged from the specialized clinic, and referred back to their Primary Care Physician for routine healthcare.*
  - ***PCFS score ≥2:*** *The patient remains under specialized surveillance and is scheduled for a follow-up consultation in 3 months.*

S2*. List of most frequent symptoms*

1. Fatigue: Yes/No
2. Asthenia: Yes/No
3. Arthralgia: Yes/No
4. Myalgia: Yes/No
5. Dyspnea: Yes/No
6. Cough: Yes/No
7. Expectoration: Yes/ No
8. Chest pain: Yes/No
9. Anosmia/Dysgeusia: Yes/No
10. Headache: Yes/No
11. Diarrhea: Yes/No
12. Insomnia: Yes/No

13. Difficulty concentrating, brain fog: Yes/No
14. Palpitations: Yes/No
15. Odynophagia: Yes/No
16. Hair loss: Yes/No
17. Vertigo: Yes/No

S3. Simple logistic regression model for persistence of post-COVID syndrome (N=112)

**Variables**		**OR**	**95% CI**	**p**
**Demographic characteristics**				
Age (years)	< 40	1.25	(0.23-6.65)	0.79
	40-54	1.70	(0.41-6.99)	0.46
	55-64	1.69	(0.36-7.88)	0.51
	≥65	Ref.		
Female sex		1.03	(0.40-2.69)	0.95
**Medical history**				
SARS-CoV-2 2 Vaccination	Incomplete or unvaccinated	1.70	(0.72-4.03)	0.22
Comorbidity	1 or more	1.20	(0.40-3.60)	0.75
Smoking	Yes	1.86	(0.62-5.60)	0.27
Hypertension	No	1.68	(0.56-5.03)	0.36
Dyslipidemia	Yes	2.05	(0.78-5.37)	0.15
Diabetes mellitus	No	1.03	(0.24-4.37)	0.97
Depression	Yes	2.55	(0.85-7.68)	0.096
Anxiety	No	1.03	(0.33-3.27)	0.96
**Acute COVID 19 Symptomatology**				
Fever	Yes	2.32	(0.96-5.59)	0.060
Cough	Yes	1.18	(0.49-2.85)	0.71
Odynophagia	Yes	1.09	(0.47-2.54)	0.84
Anosmia-ageusia	Yes	1.19	(0.54-2.62)	0.67
Headache	Yes	1.11	(0.50-2.44)	0.80
Dyspnea	No	1.02	(0.45-2.29)	0.97
Expectoration	No	1.21	(0.47-3.10)	0.70
Rhinitis	No	2.54	(0.99-6.56)	0.053
Myalgia	Yes	1.98	(0.85-4.57)	0.11
Arthralgia	No	1.62	(0.41-6.36)	0.49
Asthenia	Yes	1.24	(0.55-2.80)	0.61
Hyporexia	No	1.41	(0.61-3.29)	0.43
Diarrhea	No	1.24	(0.44-3.55)	0.68
Chest pain	Yes	1.86	(0.62-5.60)	0.27
Hospital admission	Yes	1.25	(0.47-3.34)	0.66
Infection severity	Oxygen treated	1.60	(0.61-4.23)	0.34
Corticosteroids	Yes	1.40	(0.56-3.52)	0.47
**Post-COVID symptoms (baseline visit)**				
Arthralgia	Yes	2.00	(0.89-4.46)	0.092
Cough	Yes	1.47	(0.64-3.38)	0.36
Odynophagia	Yes	1.25	(0.38-4.12)	0.71
Anosmia	No	1.19	(0.48-2.96)	0.70
Dyspnea	Yes	1.85	(0.76-4.49)	0.17
Chest pain	Yes	1.29	(0.52-3.22)	0.58
Myalgia	Yes	2.01	(0.91-4.46)	0.085
Headache	Yes	1.46	(0.66-3.20)	0.35
Insomnia	Yes	1.93	(0.87-4.28)	0.103
Brain frog	No	1.11	(0.51-2.43)	0.80
Asthenia	Yes	3.02	(1.18-7.75)	0.022
Palpitations	No	1.26	(0.53-3.03)	0.60
Fatigue	Yes	3.59	(1.50-8.62)	0.004
Hyporexia	Yes	1.29	(0.46-3.66)	0.63

Variables		OR	95% CI	p
Diarrhea	No	2.18	(0.44-10.83)	0.34
**Post-COVID baseline visit assessment**				
SARC-F	Yes	1.25	(0.57-2.74)	0.58
PCFS	Grade 3-4	3.05	(1.36-6.86)	0.007
mMRC	Grade 1	1.15	(0.20-6.70)	0.87
	Grade 2	1.50	(0.27-8.34)	0.64
	Grade 3-4	2.75	(0.46-16.59)	0.27
Depression	Mild	3.87	(0.79-18.98)	0.095
	Moderate	5.30	(1.51-18.65)	0.009
	Severe	8.23	(2.37-28.6)	0.001
Anxiety	Mild	4.00	(0.41-39.37)	0.24
	Moderate	7.87	(0.87-71.13)	0.066
	Severe	21.27	(2.63-170.3)	0.004
EQ-5D EVA	(0-100)	0.97	(0.95-0.99)	0.040
NLI	≤ P25 (2.24)	1.53	(0.58-4.04)	0.39
Time since onset of symptoms (months)		1.04	(0.98-1.09)	0.21

OR: odds ratio. 95% CI: confidence interval. Ref: reference category .SARC-F: Strength, Assistance with walking, Rise from a chair, Climb stairs, and Falls. PCFS: Post-COVID functional scale. mMRC: modified Medical Research Council dyspnea scale. EuroQol-5D (EQ-5D) VAS: Euro Quality of Life Health-Related Quality of Life Questionnaire visual analogue scale. NLI: Neutrophil-to-lymphocyte ratio.
